# Prostaglandin E2 synthesis in cartilage explants under compression: mPGES-1 is a mechanosensitive gene

**DOI:** 10.1186/ar2024

**Published:** 2006-07-27

**Authors:** Marjolaine Gosset, Francis Berenbaum, Arlette Levy, Audrey Pigenet, Sylvie Thirion, Jean-Louis Saffar, Claire Jacques

**Affiliations:** 1 UMR 7079 CNRS, Physiology and Physiopathology Laboratory, University Paris 6, quai St-Bernard, Paris, 75252 Cedex 5, France; 2 Department of Rheumatology, UFR Pierre et Marie Curie, Saint-Antoine Hospital, 75012 Paris, France; 3 CNE Neuroendocrine Cellular Interactions, UMR CNRS 6544, Mediterranean University, Faculty of Medecine, 13916 Marseille Cedex 20, France; 4 Laboratory on Oro-facial Repair and Replannings EA 2496, University Paris Descartes, Faculty of Odontology, 92120 Montrouge, France

## Abstract

Knee osteoarthritis (OA) results, at least in part, from overloading and inflammation leading to cartilage degradation. Prostaglandin E2 (PGE_2_) is one of the main catabolic factors involved in OA. Its synthesis is the result of cyclooxygenase (COX) and prostaglandin E synthase (PGES) activities whereas NAD+-dependent 15 hydroxy prostaglandin dehydrogenase (15-PGDH) is the key enzyme implicated in the catabolism of PGE_2_. For both COX and PGES, three isoforms have been described: in cartilage, COX-1 and cytosolic PGES are constitutively expressed whereas COX-2 and microsomal PGES type 1 (mPGES-1) are inducible in an inflammatory context. COX-3 (a variant of COX-1) and mPGES-2 have been recently cloned but little is known about their expression and regulation in cartilage, as is also the case for 15-PGDH. We investigated the regulation of the genes encoding COX and PGES isoforms during mechanical stress applied to cartilage explants. Mouse cartilage explants were subjected to compression (0.5 Hz, 1 MPa) for 2 to 24 hours. After determination of the amount of PGE_2 _released in the media (enzyme immunoassay), mRNA and proteins were extracted directly from the cartilage explants and analyzed by real-time RT-PCR and western blotting respectively. Mechanical compression of cartilage explants significantly increased PGE_2 _production in a time-dependent manner. This was not due to the synthesis of IL-1, since pretreatment with interleukin 1 receptor antagonist (IL1-Ra) did not alter the PGE_2 _synthesis. Interestingly, COX-2 and mPGES-1 mRNA expression significantly increased after 2 hours, in parallel with protein expression, whereas COX-3 and mPGES-2 mRNA expression was not modified. Moreover, we observed a delayed overexpression of 15-PGDH just before the decline of PGE_2 _synthesis after 18 hours, suggesting that PGE_2 _synthesis could be altered by the induction of 15-PGDH expression. We conclude that, along with COX-2, dynamic compression induces mPGES-1 mRNA and protein expression in cartilage explants. Thus, the mechanosensitive mPGES-1 enzyme represents a potential therapeutic target in osteoarthritis.

## Introduction

Osteoarthritis (OA) is the leading cause of disability among the elderly population [1]. Traumatic joint injury and joint overload are two major causes of cartilage degradation leading to OA. Although the process of this disease is not yet fully understood, it results from an imbalance in the loss of cartilage caused by matrix degradation and the death of the unique cellular population of cartilage, the chondrocytes. Joints are physiologically exposed to mechanical stress, which triggers gene expression and metabolic activity of chondrocytes in order to turn over the extra cellular matrix and eventually adapt the tissue to loading. The magnitude of the forces that are physiologically applied to cartilage is up to 20 MPa, according to the type of articulation, movement and weight of the individual [2]. Moreover, pressure that is applied on joints comprises a complex combination of strain, shear stress and compressive forces, the latter seemingly being more prevalent in cartilage. The duration of mechanical stress is less than 1 second and leads to cartilage deformation of only 1% to 3% [3]. Many biochemical changes are associated with cartilage degradation and OA progression. These include an increased production of matrix metalloproteinases, proinflammatory cytokines, proinflammatory lipid mediators, extracellular nucleotides, reactive oxygen species and reactive nitrated oxygen species as nitric oxide (NO). It is noteworthy that abnormal cartilage loading may trigger the synthesis of all of these mediators [4-6]. Notably, Fermor and colleagues [6] described that intermittent compression (0.5 Hz, 24 hours, 0.1 to 0.5 MPa) caused an increase in NO production and inducible NO synthase activity (P < 0.05). Different mechanoreceptors have been proven to be at the surface of chondrocytes [7], but the integrin α5β1 could be the major link between extracellular mobilization and intracellular events [8], which eventually promote the synthesis of the various mediators described above. Recent studies have focused on the intracellular events that promote these syntheses under mechanical stress. Among them are the extracellular signal regulated kinases 1/2 (ERK1/2), p38 mitogen-activated protein kinase (p38) and c-jun-N-terminal kinase (JNK) [9], known for their involvement in many biological events.

Prostaglandin E2 (PGE_2_) is one of the major catabolic mediators involved in cartilage degradation and chondrocyte apoptosis [10-12]. OA cartilage spontaneously releases more PGE_2 _than normal cartilage [13] and in knock-out mice for EP4, a membrane receptor for PGE2, a decreased incidence and severity of cartilage degradation in the collagen-induced arthritis model is observable [14]. Several studies have examined the effects of physical forces on PGE_2 _release. On the one hand, cyclic tensile strain [15] and dynamic compression applied on chondrocytes cultured in agarose for 48 hours [16] inhibited the release of PGE_2. _On the other hand, fluid-induced shear stress [17] as intermittent mechanical compression for 1 hour increased PGE_2 _release in chondrocytes [6]. So, depending on the type, the magnitude and duration of mechanical stress, different molecular events, such as PGE_2 _release, are triggered in chondrocytes.

PGE_2 _is a prostanoid derived from arachidonic acid released from membranes by phospholipase A_2_. Arachidonic acid is metabolized by cyclooxygenase (COX) activity to form the prostaglandin endeperoxyde H_2_. Three isoforms of COX (COX-1, COX-2 and COX-3) have been cloned. Whereas COX-1 is constitutively expressed in various cell types to maintain homeostasis, COX-2 is inducible in an inflammatory environment. COX-3 is a recently described derivative of COX-1 that occurs as the result of conservation of the first intron and is also called COX-1 V1. At this time, its expression is described in both canine and human cortex and aorta, and in the rodent heart, kidney and neuronal tissues [18]. Prostaglandin endeperoxyde H_2 _is subsequently metabolized by PGE synthase (PGES) to form PGE_2_. Three types of PGES have been cloned. The cytosolic form (cPGES) is ubiquitous and non-inducible, whereas the microsomal PGES type 1 (mPGES-1) is involved in PGE_2 _synthesis during inflammation. mPGES-1-deficient mice exhibit a significant reduction in disease severity and cartilage degradation in the collagen-induced arthritis model [19,20]. mPGES-1 belongs to the MAPEG family (membrane associated proteins in eicosanoid and glutathion metabolism) and is glutathion dependent. We and others have recently shown that IL-1β upregulates mPGES-1 expression in OA chondrocytes [21,22]. A third form of PGES, called microsomal PGES type 2 (mPGES-2), has recently been cloned. This glutathion-independent enzyme is expressed in various cells and seems to be poorly regulated by inflammation [23]; however, its expression and regulation have not yet been elucidated in cartilage.

Our investigation sought to explore the activation of the arachidonic acid cascade. We hypothesized that mechanical compression in certain conditions would lead to PGE_2 _synthesis by chondrocytes. Furthermore, we wanted to determine whether genes encoding COX and PGES isoforms are mechanosensitive or not.

## Materials and methods

### Materials

All of the reagents were purchased from Sigma-Aldrich (St Quentin Fallavier, France), unless stated otherwise. Collagenase D and a Complete protease inhibitor mixture were from Roche Diagnostics (Meylan, France). Antibodies used were: anti-mouse COX-2 polyclonal antibody (Santa Cruz Biotechnology from Tebu, Le Perray-en-Yvelines, France); anti-mouse COX-3 polyclonal antibody (Alpha Diagnostic International, San Antonio, Texas, USA); anti-mouse COX-1 polyclonal antibody; anti-mouse mPGES-1 polyclonal antibody; anti-mouse mPGES-2 polyclonal antibody; anti-mouse cPGES polyclonal antibody (Cayman from SPI-BIO, Massy, France); and anti-mouse β-actine monoclonal antibody. The ECL western-blot analysis system was purchased from Amersham Pharmacia Biotech (Orsay, France). The Immuno-Blot polyvinylidene difluoride (PVDF) membranes for western-blotting and kaleidoscope prestained standards were obtained from Bio-Rad (Ivry-sur-Seine, France). Inteleukin 1 receptor antagonist (IL1-Ra) was obtained from R&D Systems (Minneapolis, MN, USA). Anti-goat fibronectin receptor (integrin α5β1) blocking polyclonal antibody (AB1950) was purchased from Euromedex for Chemicon Inc. (Strasbourg, France) and rat anti-mouse β1 subunit of VLA1 integrins non-blocking monoclonal antibody (VMA 1997), was purchased from AbCys SA for Chemicon Inc. (Souffelweyersheim, France).

### Compression experiments

**Figure 1:**
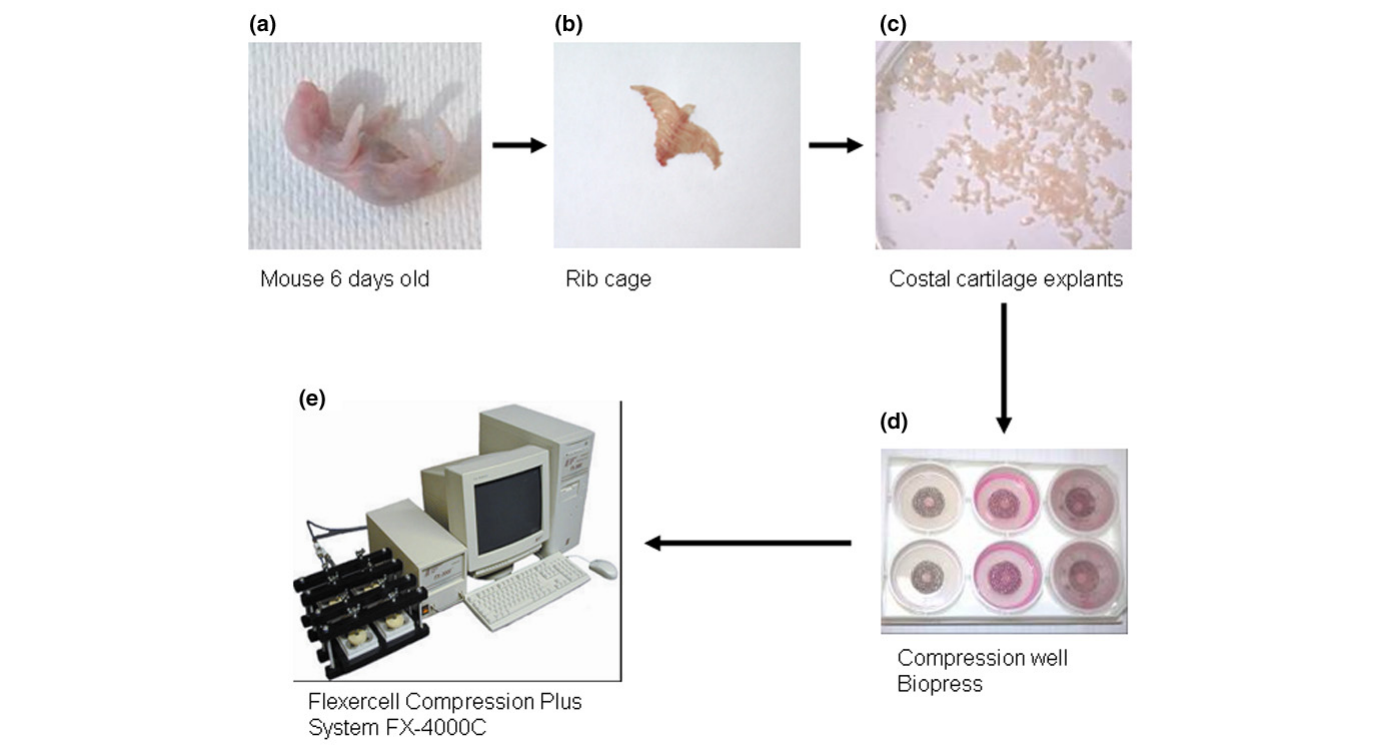
Mouse cartilage explants and Flexercell apparatus employed for mechanical stimulation. **(a,b) **Rib cages were harvested from one litter of 6-day-old Swiss mice. **(c) **Costal cartilage was cleaned and cut into little segments. 50 mg of the costal cartilage pool were put into a Biopress culture plate and 1.5 ml of media was added. **(d) **Each well was hermetically sealed with a specific cap. **(e) **The physiological compressive stress was applied by the Flexercell Compression Plus system described by Fermor and colleagues [26] on mouse costal cartilage explants. Intermittent compression was applied using a sinusoidal waveform at 0.5 Hz and 1.0 MPa of magnitude.

All of the experiments were performed according to the protocols approved by the French/European ethics committee. Compression was applied either on costal cartilage or on articular catilage. For each experiment, all of the rib cages and all of the knees and the hips were harvested from 6-day-old newborns from one Swiss mouse litter according to the procedure described in [24,25] (Figure 1).

For costal cartilage, explants were cleaned in PBS to eliminate soft tissues and bone sternum parts were discarded. The costal cartilage was cut and divided into segments, which were pooled. Each sample consisted of 50 mg of costal cartilage explants. For articular cartilage, cartilage of two femoral heads and two knees constitute one sample.

Immediately after the dissection, each sample was placed into individual compression wells of Biopress culture plates (Flexercell International, Hillsborough, NC, USA) in 1.5 ml of culture medium (DMEM, containing penicillin-streptomycin 1% v/v, glutamin 2% v/v, albumin 0.1% v/v and Hepes 30 mM) (Figure 1). All of the experiments were performed at 37°C, in air. The compressive stress was applied to individual samples by the Biopress system (Flexercell International) described by Fermor and colleagues [26], whereas the control explants were kept in unloaded conditions. At each time point (2 h, 4 h, 18 h and 24 h), we analyzed compressed and uncompressed explants supplemented or not with effectors. Our results are expressed as fold-induction in comparison to controls. After the application of the mechanical regimen, supernatants and cartilage explants were collected and stored immediately at -20°C and -80°C, respectively.

Intermittent compression was applied using a sinusoidal waveform at 0.5 Hz (1 s on, 1 s off) for 30 minutes to 24 hours. Fermor and colleagues [26] have established a calibration graph for the Biopress system. This calibration establishes a linear relationship between air pressure and the corresponding compression force applied on a 5 mm diameter cartilage disk. This calibration was calculated on a cross-sectional area of the explant. In our model, the cartilage explants were disposed of in order to form a 5 mm disk, which was composed of several cartilage explants. We considered that the mechanical stress applied is less uniform, but still is 1.0 MPa for an air pressure of 30 kPa, according to the calibration from Fermor and colleagues [26].

### Cell viability assay

Immediately after compression, cartilage was first incubated with collagenase D solution (3 mg/ml) for 90 minutes at 37°C, and then incubated with collagenase D (0.5 mg/ml) overnight at 37°C. The cell suspension obtained was mixed to disperse any cell aggregates, producing a suspension of isolated cells. Cells suspended in a culture medium were colored with Trypan blue (0.04%) and counted in a hemocytometer. This cell viability assay was carried out on one uncompressed and two compressed explants, at 4 hours and 24 hours, and on one explant immediately after the dissection, in two independent experiments.

### PGE_2 _and NO assays

Absolute concentrations of nitrite, a stable end-product of NO metabolism, were determined in the media of the cartilage explants using a spectrophotometric method based on the Griess assay (Griess Reagent System, Promega, Charbonnières, France). Absorbance was measured at 550 nm and nitrite concentration was determined by comparison with standard solutions of sodium nitrite.

PGE_2 _production was measured in the media by a high sensitivity commercially available enzyme immunoassay kit (Cayman Chemical, Ann Arbor, MI, USA), as previously described [27]. The cross-reactivity of the antibody with other prostanoids is 43% PGE_3_, 37.4% 8-*iso *PGE_2_, 18.7% PGE_1_, 1% PGF_1_α and 0.25% 8-*iso *PGF_2_α. The limit of detection was 9 pg/ml. PGE_2 _concentration was analyzed at serial dilutions in duplicate and was read against a standard curve.

### RNA extraction, reverse transcription and quantitative real-time PCR

Frozen cartilage explants (50 mg) were milled in 600 μL of RLT buffer (from RNeasy Mini Kit, Qiagen GmbH, Hilden, Germany) using a Mixer Mill MM 300 apparatus (Qiagen). Disruption was achieved through the beating and grinding effect of beads on the cartilage samples as they were shaken together in the grinding vessels. One steel ball (diameter 5 mm) was added to each sample and they were mixed, at a cool temperature, for two cycles of 2 minutes at 25 pulses/second. Then, after removing the beads, the total RNA was extracted from each sample using the RNeasy Kit (Qiagen) according to the manufacturer's instructions. A proteinase K (Qiagen) digestion step was performed after the lysis of cartilage explants and a DNAse digestion step (RNAse free DNAse set, Qiagen) was added. RNA concentration was then measured using a spectrophotometer. The migration in an agarose gel enabled quality control.

Total RNA (1 μg) was reverse transcribed with Omniscript (Qiagen) in a final volume of 20 μL containing 50 ng of oligos dT. The enzyme was then inactivated by heating and the interesting mRNAs (COX-1, genbank BC005573; COX-2, NM_011198; COX-3, AY547265; mPGES-1, NM_022415; mPGES-2, BC004846; cPGES, NM-008278; 15-PGDH, NM_008278) were quantified by real-time quantitative reverse transcription RT-PCR using the iCycler iQ Real Time PCR (Bio-Rad) and QuantiTect SYBR PCR kits (Qiagen). Sense and antisense PCR primers were designed based on mouse sequence information for the amplification of genes of interest (Table 1). The PCR reactions were performed in a 25 μl final volume using 0.06 to 0.25 μl of cDNA, 600 ng of specific primers and 1× QuantiTect SYBR Green PCR master mixture, including HotStar *Taq *DNA Polymerase, QuantiTect SYBR Green PCR buffer, SYBR Green I, and ROX in which there was 5 mM MgCl. PCR amplification conditions were: initial denaturation for 13 minutes at 95°C followed by 50 cycles consisting of 30 seconds at 95°C and 30 seconds at 58°C. Product formation was detected at 72°C in the fluorescein isothiocyanate channel. The generation of specific PCR products was confirmed by melting-curve analysis. For each real-time RT-PCR run, cDNA were run in quadruplicate in parallel with serial dilutions of a cDNA mixture tested for each primer pair to generate a linear standard curve, which was used to estimate relative quantities of COX, PGES and 15-PGDH mRNA normalized for Hypoxanthine-guanine phosphoribosyltransferase (HPRT genbank NM_008278) in the samples.

### Protein extraction and western blotting

Frozen cartilage explants were disrupted using a Mixer Mill MM 300 apparatus (Qiagen) in 500 μL of cold lysis buffer (20 mM Tris pH 7.6, 120 mM NaCl, 10 mM EDTA, 10% glycerol, 1% Nonidet P-40, 100 mM NaF; 10 mM Na_4_P_2_0_7_, 1 mM AEBSF (4-(2-Aminoethyl)benzenesulphonyl fluoride), 2 mM Na_3_VO_4_, 40 μg/ml leupeptin, 1 μM pepstatin A, 10 μg/ml aprotinin). One steel ball (diameter 5 mm) was added to each sample, which were mixed at a cool temperature for two cycles of 2 minutes at 25 pulses/second. Then, after removing the beads, the samples were shaken gently for 1 hour at 4°C and then centrifuged for 1 hour (13,000 g, 4°C). The supernatants were collected and protein concentrations were determined using the bicinchoninic acid assay kit (Perbio Science for Pierce, Bezons, France).

Cartilage explant lysates were separated by 8% or 15% SDS-PAGE and transferred to nitrocellulose membranes. The blots were incubated (then stripped and reprobed) by the appropriate primary polyclonal antibody to COX-2, COX-3, COX-1, mPGES-1, mPGES-2, cPGES and monoclonal antibody to β-actin. The blots were then incubated with horseradish peroxidase-conjugated secondary goat antibody. The membranes were washed repeatedly with Tris-buffered Saline containing Tween-20 0.1% (v/v) and the signals were detected using the enhanced chemiluminescence detection system and exposed to Kodak BioMax MR-1 film. We transfected Cos cells with plasmids encoding COX-2 and mPGES-1. Cells extracts containing COX-2 and mPGES-1 proteins surexpressed were used as positive controls.

### Immunohistochemistry

After compression for 18 hours, cartilage explants were immediately collected and fixed in 70% ethanol at 4°C for 48 hours. After dehydratation, the cartilage samples were embedded without demineralization in methyl methacrylate (Merck, Darmstadt, Germany). Transversal sections (4 μm thick) were cut parallel to the rib axis using a Polycut E microtome (Leica, Wetzlar, Germany). Sections mounted onto slides were deplastified in 2-methoxyethylacetate prior to further processing. The primary polyclonal antibodies used were the same as those used for western blotting, as previously described. For immunochemistry, the sections were incubated overnight with 0.1 M PBS supplemented with 0.05% Tween 20 (Sigma) and 1% BSA (Euromedex) and the primary polyclonal antibody (1:50) at 4°C in a moist chamber. The sections were then incubated with biotinylated goat anti-rabbit IgG for PGES or rabbit anti-goat IgG for COX-2 (Vector, Burlingame, CA, USA) for 90 minutes at room temperature. They were then treated with 3% hydrogen peroxide (10 minutes), and an avidin-biotin peroxidase complex (ABC Vectastain kit, Vector) for 60 minutes. PBS (0.1 M) was used for the washing steps between incubations. Diaminobenzidine tetrahydrochloride (Sigma) was used as the chromogen. The sections were lightly counterstained with toluidine blue (pH 3.8). Negative controls were prepared by omitting the primary antibody in the diluant solution (BSA 1% and goat serum 10% for PGES, and BSA 10% and milk 1% for COX-2). Immunohistological analysis was carried out on two uncompressed and two compressed costal cartilage explants. Images were obtained using an optical microscope and analysis for each enzyme utilized a blind test.

### Statistical analysis

All data are reported as mean ± SEM, unless stated otherwise. Unpaired Students' t-tests were used to compare the mean values between groups with the GraphPad InStat version (GraphPad Software, San Diego, California, USA). The P values ≤ 0.05 are considered to be significant.

## Results

### Compressive stress triggers the synthesis of NO and PGE_2 _via α5β1 integrin but not via IL-1 synthesis

**Figure 2:**
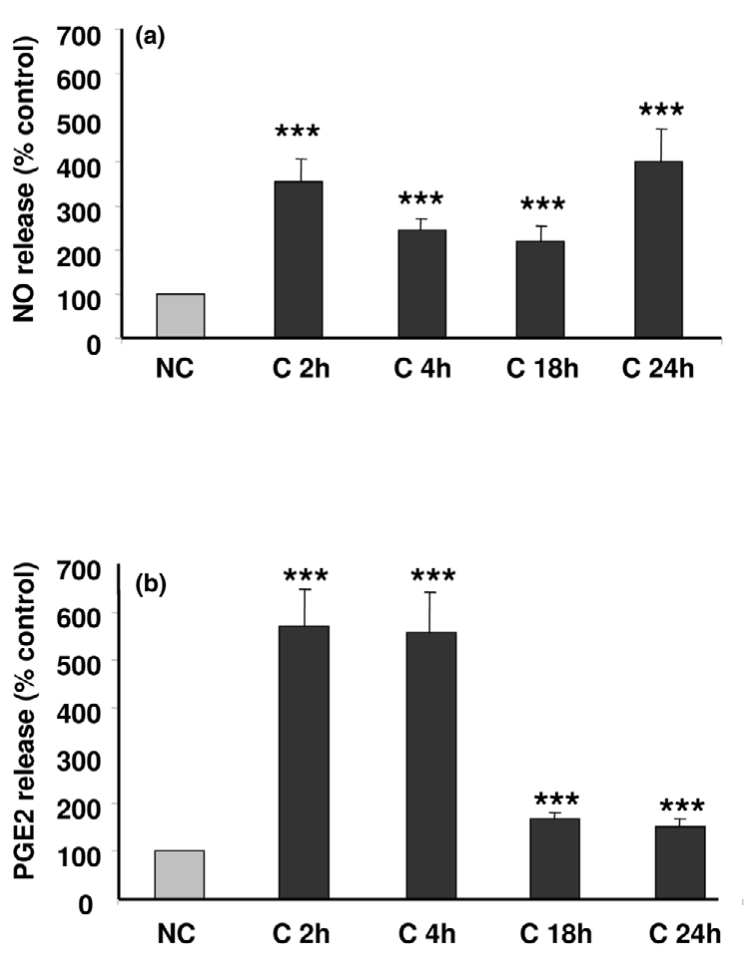
Compression stimulates nitric oxide (NO) and prostaglandin E2 (PGE_2_) release in mouse costal cartilage explants in the media. Mouse costal cartilage explants were compressed (C) or not (NC) for 2 h, 4 h, 18 h and 24 h. At each time interval, our results are expressed in fold-induction in comparison to the appropriate control. **(a) **The amount of NO released into the media (μmol/mg of costal cartilage) was measured by Griess reagent. Values are the mean and SEM of 3 (C 2 h and 4 h) and 2 (C 18 h and 24 h) independent experiments with *n *= 2/group/experiments. ***p < 0.001 versus control (NC). **(b) **The amount of PGE_2 _released into the media (pg/mg/ml of costal cartilage) was measured by enzyme immunoassay. Values are the mean and SEM of 3 (C 2 h), 2 (C 4 h and 18 h) and 4 independent experiments (C 24 h) with *n *= 2/group/experiments, analyzed in duplicate. ***p < 0.001 versus control (NC).

To determine the effects of compressive stress on chondrocyte activation, we assessed NO and PGE_2 _release in the media in compressed and uncompressed costal cartilage explants. Different magnitudes and lengths of stress were applied in order to define the optimum conditions (data not shown). At a sinusoidal waveform frequency of 0.5 Hz and a magnitude of 1 MPa, NO release significantly increased (6-fold increase; p < 0.001) within 2 hours compared to uncompressed explants, and lasted 24 hours (Figure 2a), as described by Fermor and colleagues [6]. Also, PGE_2 _synthesis in the media significantly increased, with a peak at 2 hours that was sustained up to 4 hours (6-fold increase; p < 0.001) and then decreased at 24 hours (Figure 2b).

**Figure 3:**
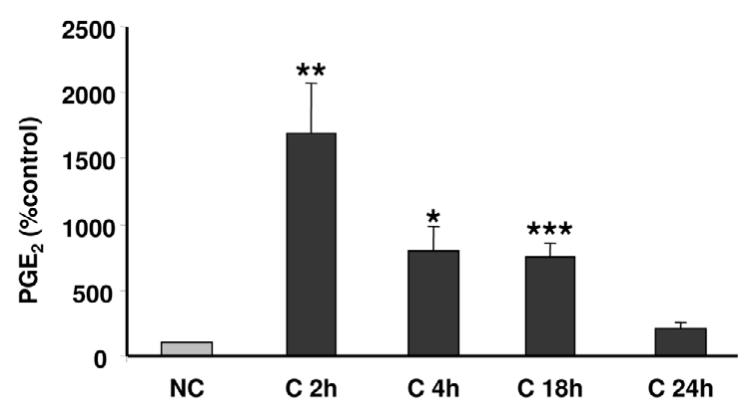
Compression stimulates prostaglandin E2 (PGE_2_) release in mouse articular cartilage explants. Mouse articular cartilage explants were compressed (C) or not (NC) for 2 h, 4 h, 18 h and 24 h. The amount of PGE_2 _released into the media (pg/ml) was measured by enzyme immunoassay. Values are the mean ± SEM of 2 independent experiment with *n *= 2/group/experiments, analyzed in duplicate. *p < 0.05, **p < 0.01, ***p < 0.001 versus control (NC).

Compressive stress was also applied to mouse articular cartilage explants in order to avoid a bias due to the origin of cartilage. As in costal cartilage, articular cartilage explants submitted to compression exhibit an increase in PGE_2 _release after 2 hours (16-fold increase; p < 0.01), which was sustained from 4 hours to 18 hours (6-fold increase) before declining to control levels. Even though PGE_2 _release in articular cartilage (16-fold increase, p < 0.01) was stronger at 2 hours than in costal cartilage (6-fold increase, p < 0.001) and was sustained for longer, only minor differences in kinetics were observed (Figure 3).

Viability of the chondrocytes in the mouse costal cartilage explants was tested using Blue Trypan coloration. No alteration of cell viability was seen between compressed and uncompressed samples within 24 hours (data not shown).

**Figure 4:**
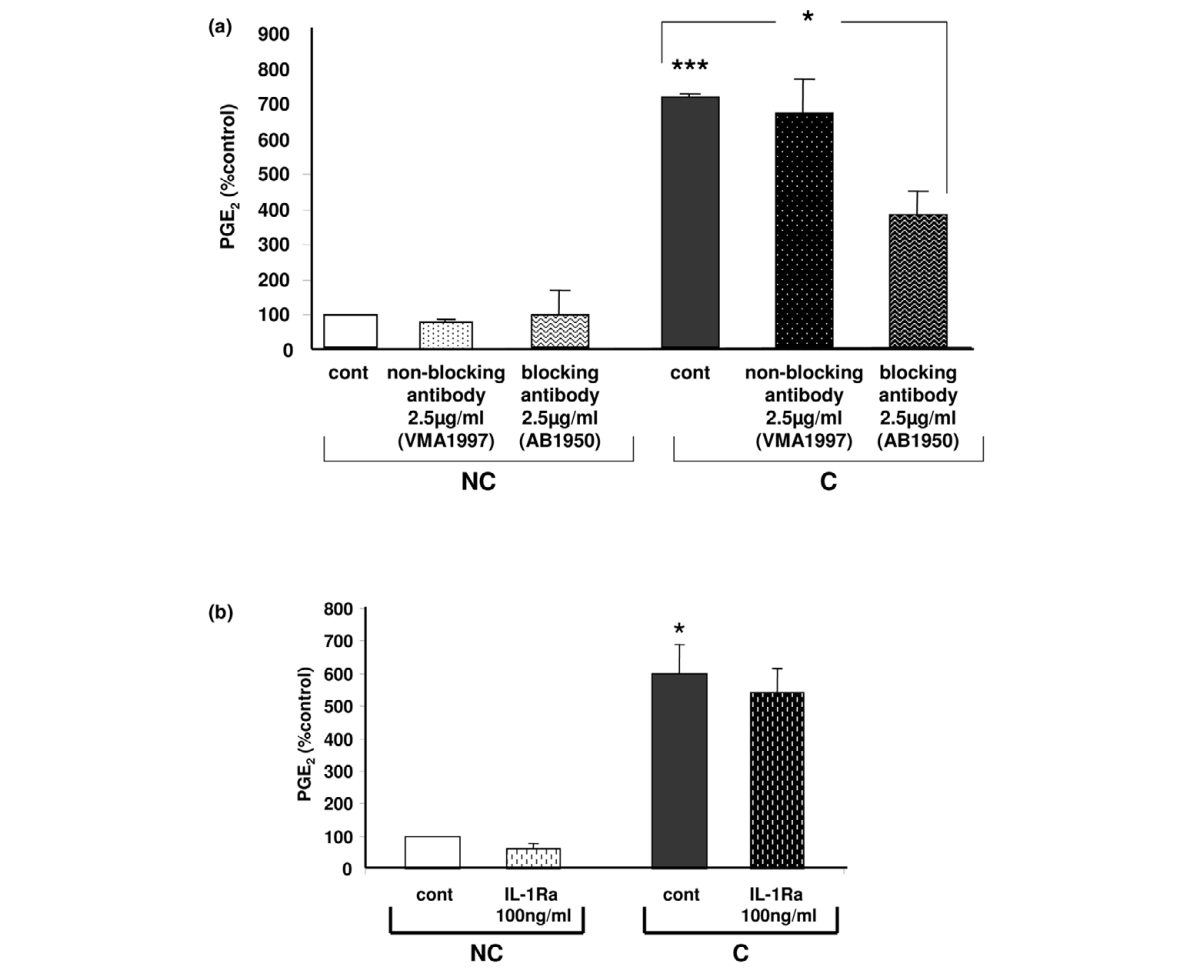
Over-release of prostaglandin E2 (PGE_2_) in compressed costal cartilage explants is the result of mechanical stress. **(a) **Implication of the mechanoreceptor integrin α5β1 in PGE_2 _over-release in compressed cartilage explants. Mouse costal cartilage explants treated with either the β1 non-blocking antibody VMA1997 or the α5β1 blocking antibody AB1950 at 2.5 μg/ml were compressed (C) or not compressed (NC) for 4 h. Results are normalized to the mean not-compressed control (cont) value. Data are the mean ± SEM of 2 independent experiments with *n *= 2/group/experiments, analyzed in duplicate. ***p < 0.001 versus control NC, *p < 0.05 versus control C. **(b) **Increased PGE_2 _release in compressed costal cartilage explants is not due to the cytokine IL-1. Mouse costal cartilage explants treated with the IL-1 receptor antagonist (IL1-Ra) at 100 ng/ml were compressed (C) or not compressed (NC) for 4 h. Results are normalized to the mean not compressed control value. Data are the mean ± SEM of 2 independent experiment with *n *= 2/group/experiments, analyzed in duplicate. *p < 0.05 versus control NC.

To confirm the validity of our compressive model on mouse costal cartilage, we wanted to highlight the implication of integrin α5β1 in the PGE_2 _release triggered by mechanical stress. Cartilage explants treated with anti-integrin α5β1 blocking antibody (AB1950) at 2.5 μg/ml induced a 50% decrease of compression-induced NO (4.16 ± 0.57 versus 2.68 ± 0.43 μM; data obtained from 2 independent experiments with *n *= 2/group/experiments; p < 0.001, data not shown). Moreover, a decrease in PGE_2 _release of approximately 50% in compressed cartilage treated with the blocking α5β1 antibody was observed (Figure 4a). No modification in NO (4.01 ± 0.1 versus 4.4 ± 1.43 μM; data obtained from 2 independent experiments with *n *= 2/group/experiments, data not shown) or PGE_2 _release (Figure 4a) was detected in media of compressed cartilage treated with the non-blocking anti-β1 subunit antibody at 2.5 μg/ml (VMA1997).

We previously reported that the pro-inflammatory cytokine IL-1 triggers the expression of COX-2 and mPGES-1 [21,28]. Since the integrin antibody did not fully inhibit a compression-induced PGE_2 _release, even if other mechanoreceptors have been described on chondrocytes, we hypothesized that compression could indirectly act on cartilage by inducing the synthesis of IL-1. When the IL-1 receptor antagonist IL1-Ra was added at a concentration of 100 ng/ml prior to compression, no variation in PGE_2 _release was observed (Figure 4b), suggesting that compression-induced PGE_2 _release is not mediated by IL-1.

### Expression of COX and PGES enzymes in uncompressed and compressed cartilage explants

**Figure 5:**
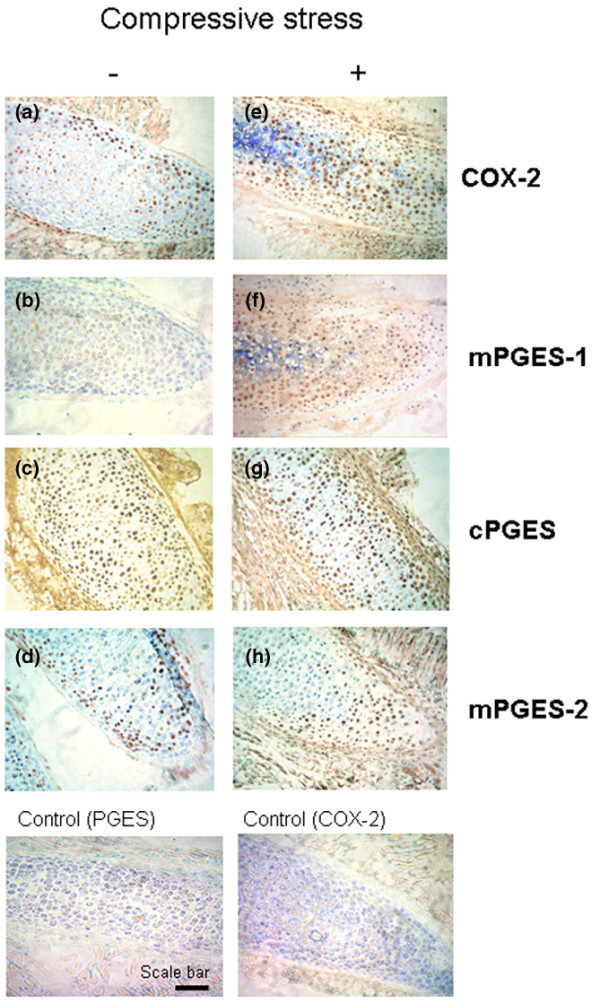
Compression increases cyclooxygenase type 2 (COX-2) and microsomal prostaglandin E synthase type 1 (mPGES-1) but not cytosolic PGES (cPGES) and mPGES-2 protein expression in costal cartilage. Costal cartilage explants were **(a-d) **not compressed or **(e-h) **compressed for 18 h and immunostained with anti-COX-2, anti-mPGES-1, anti-cPGES and anti-mPGES-2 antibodies and then counterstained with toluidine blue. Increased expression of **(e) **COX-2 and **(f) **mPGES-1 protein was seen in compressed explants compared to uncompressed (**(a) **COX-2 and **(b) **mPGES-1). In contrast, **(g) **cPGES and **(h) **mPGES-2 were not overexpressed after application of a compressive stress compared to the uncompressed condition (**(c) **cPGES and **(d) **mPGES-2). Representative findings from two compressed and two uncompressed samples were tested. Scale bar = 100 μM.

We subsequently focused our study on the enzymes involved in PGE_2 _synthesis, cyclooxygenases and prostaglandin E synthases. Mouse costal cartilage explants subjected to compressive stress for 18 hours were fixed in ethanol, embedded in methyl methacrylate and cut into serial sections that were immunostained with antibody against COX-2, mPGES-1, mPGES-2 and cPGES. Toluidine blue counterstaining colors the extracellular matrix and nuclei of cells. In uncompressed cartilage, a few peripheral cells presented positive immunostaining (brown) for COX-2 and none did so for mPGES-1. After compression, an increased brown staining for COX-2 and mPGES-1 in cells was visible around the nuclei, suggesting a colocalization of these enzymes in the perinuclear region in loaded chondrocytes. For cPGES and mPGES-2, no differences appeared in chondrocytes from compressed cartilage explants compared to uncompressed explants (Figure 5).

### COX expression in cartilage explants subjected to compression

**Figure 6:**
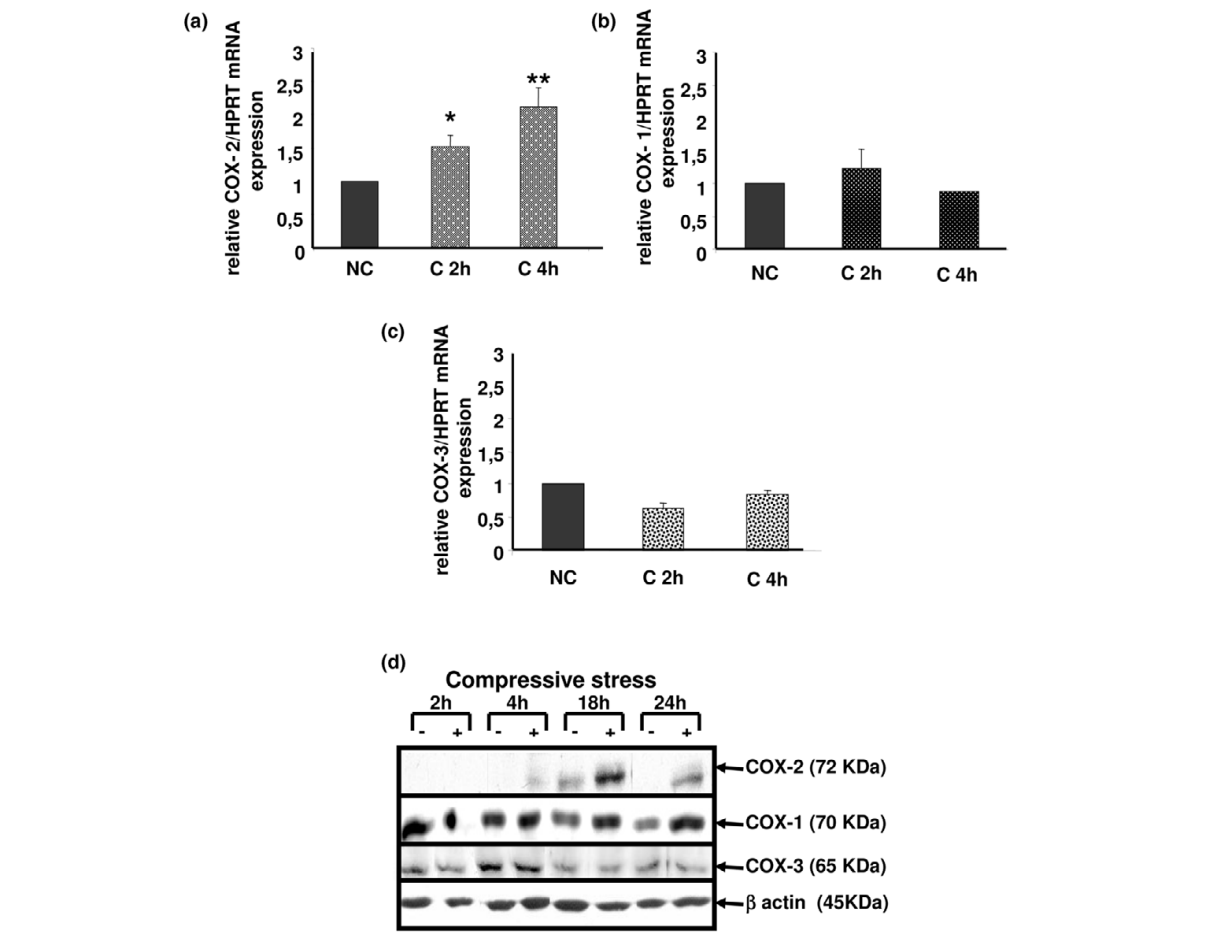
Compression increases cyclooxygenase type 2 (COX-2) gene expression but not COX-1 nor COX-3 in mouse costal cartilage explants. **(a-c) **Real-time RT-PCR assays demonstrating increased COX-2 gene expression after 2 h and 4 h in compressed explants versus control and no increase for COX-1 and COX-3. Standard curves for COX-1, COX-2, COX-3 and hypoxanthine-guanine phosphoribosyltransferase (HPRT) were generated by serial dilution of a cDNA mixture. The amount of COX-1, COX-2 and COX-3 mRNA was normalized against the amount of HPRT mRNA measured in the same cDNA. Values are the mean ± SEM of 4 independent experiments with *n *= 1/group/experiment for COX-1 and COX-2 and of 2 independent experiments with *n *= 1/group/experiment for COX-3. *p < 0.05, **p < 0.01 versus control (NC). **(d) **Explant lysates were analyzed by SDS-PAGE using 8% gradient gels. Proteins were transferred to a nylon membrane and successively blotted with anti-COX-1, anti-COX-2, anti-COX-3 and anti-β-actin antibodies. An increased expression of COX-2 protein in compressed cartilage compared to uncompressed, but not COX-1 and COX-3, was observed after 4 hours of compression up to 24 hours. Each blot is representative of three independent experiments.

To study the effects of mechanical loading on COX gene expression, we used real-time RT-PCR quantitative analysis and immunoblotting to evaluate, respectively, the transcriptional and translational expression of COX-1, COX-2 and COX-3 genes. We extracted the total RNA and proteins directly from costal cartilage explants. An increased expression of COX-2 mRNA, but not COX-1, was observed after 2 hours with a maximal effect after 4 hours of compression (Figure 6a,b). Interestingly, COX-3 mRNA was expressed in cartilage but compressive stress had no effect on its transcriptional expression in cartilage explants (Figure 6c).

We then assessed COX protein levels by immunoblotting using polyclonal antibodies raised against COX-1, COX-2 and COX-3. As expected, compression induced COX-2 protein expression after 4 hours and peaked at 18 hours, whereas COX-1 expression remained unchanged. Both the COX-3 protein and its mRNA were expressed in cartilage; however, compression did not modify its expression (Figure 6d).

### PGES expression in cartilage explants under compression

**Figure 7:**
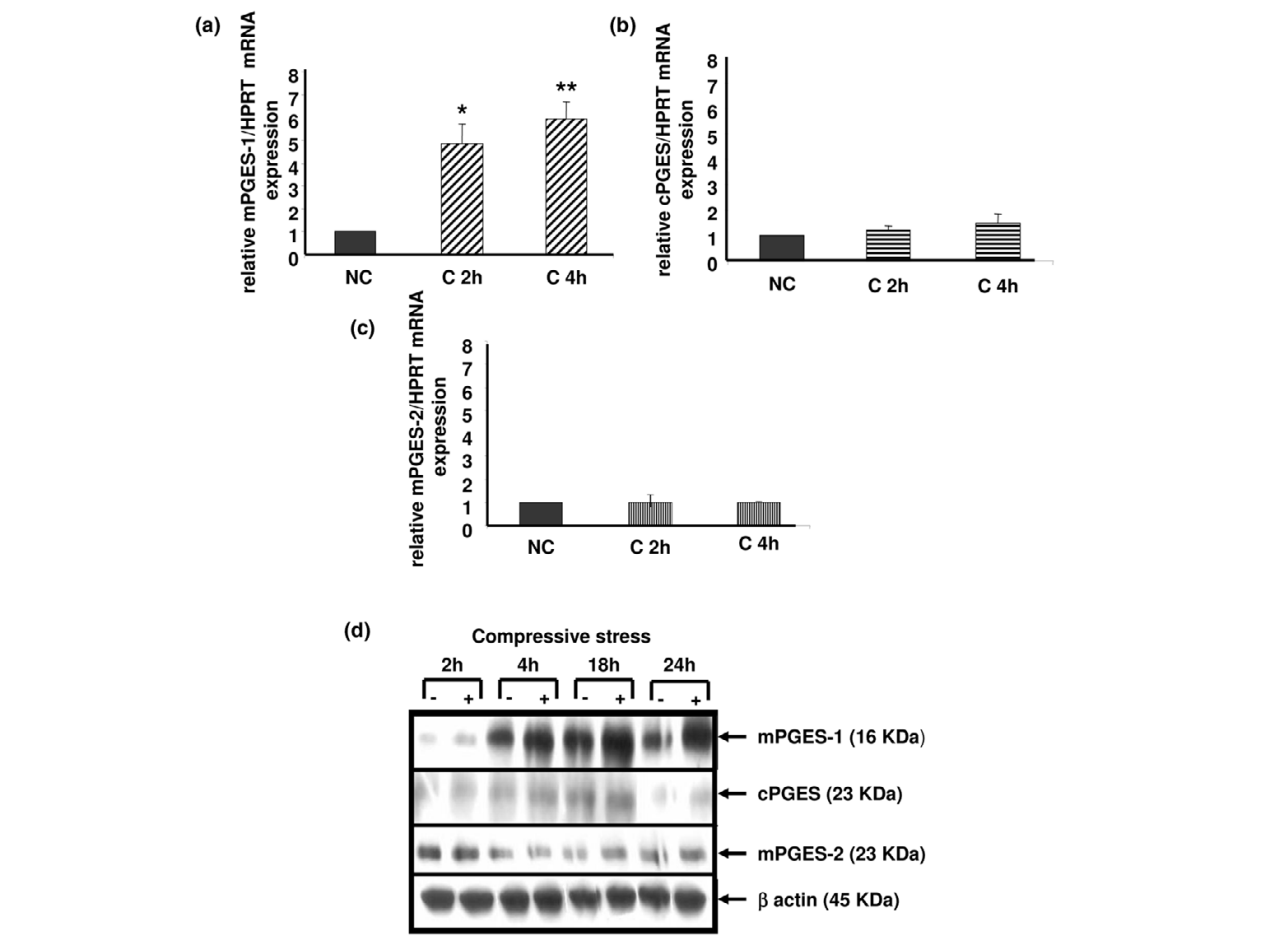
Compression increases microsomal prostaglandin E synthase type 1 (mPGES-1) gene expression but not mPGES-2 nor cytosolic PGES (cPGES) in mouse costal cartilage explants. The rates of mPGES-1, cPGES and mPGES-2 expression in response to compressive stress at 2 h and 4 h were analyzed by **(a-c) **real-time quantitative RT-PCR and **(d) **immunoblotting. **(a-c) **Up-regulation of mPGES-1 mRNA expression but not cPGES and mPGES-2 mRNA expression by mechanical stress appeared at 2 hours until 4 hours. Values are the mean ± SEM of 3 independent experiment with *n *= 1/group/experiment. *p < 0.05, **p < 0.01 versus control (NC). **(d) **Increased translational expression of mPGES-1 but not cPGES and mPGES-2 was observed on the immunoblot (15% gradient gels), from 4 hours until 24 hours. Each blot is representative of three independent experiments.

Expression of mPGES-1 but not cPGES mRNA increased after 2 hours of compression with a peak at 4 hours. Similarly, the amount of mPGES-1 protein increased in compressed explants after 2 hours, peaking at 18 hours (Figure 7a,b,d). The increased expression over the time of mPGES-1 protein in uncompressed samples, which was also observed for COX-2, could be triggered by mediators release during explantation and cutting of cartilage.

Interestingly, mPGES-2 was not regulated by compressive stress, both at the mRNA and protein levels (Figure 7c,d).

### The gene encoding 15-prostaglandin dehydrogenase is mechanosensitive

Because our results highlighted a discrepancy between the kinetics of PGE_2 _release and COX-2 and mPGES-1 expression (compare Figure 2b with Figures 6 and 7), we hypothesized that the decrease in PGE_2 _production observed after 4 hours was due, at least in part, to the activation of a catabolic pathway of PGE_2_.

**Figure 8:**
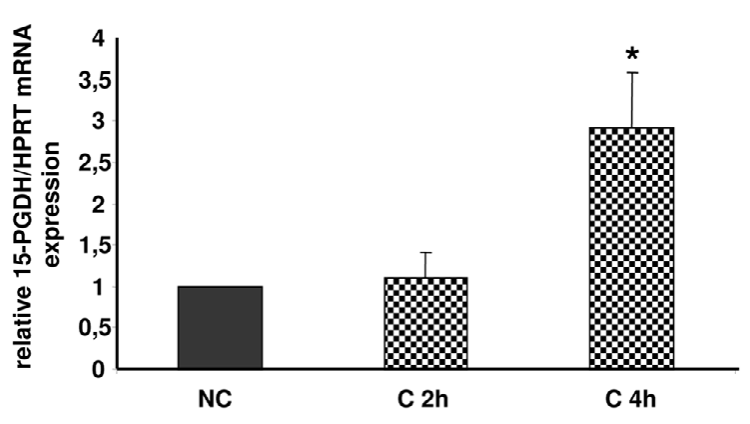
Compression increases NAD+-dependent 15 hydroxy prostaglandin dehydrogenase (15-PGDH) transcriptional expression in mouse costal cartilage explants. The rate of 15-PGDH mRNA expression in response to a compressive stress (C) at 2 hours and 4 hours was analyzed by real-time quantitative RT-PCR. Increased 15-PGDH mRNA expression was observed after 4 hours of a compressive stress. Values are the mean ± SEM of 3 independent experiment with *n *= 1/group/experiment. *p < 0.05 versus control (NC).

NAD+-dependent 15 hydroxy prostaglandin dehydrogenase (15-PGDH) is considered to be the key enzyme in the catabolism of PGE_2_. Interestingly, the gene encoding 15-PGDH is mechanosensitive and the kinetics of its expression is in agreement with our hypothesis since a peak of expression is observed at 4 hours (Figure 8).

## Discussion

Our findings demonstrate that dynamic mechanical loading of costal cartilage can significantly increase PGE_2 _release. Moreover, we describe here for the first time that COX-2 and mPGES-1 expression is increased in mouse costal cartilage explants under compression but not their constitutive isoforms COX-1 and cPGES. Therefore, it appears that COX-2 and mPGES-1 are encoded by mechanosensitive genes implicated in the compression-induced PGE_2 _release. PGE_2 _is the pivotal eicosanoid involved in the initiation and the development of inflammatory disease, such as rheumatoid arthritis [29]. Notably, it is thought to be a key regulator of cartilage degradation during OA [30]. An increase in PGE_2 _release induced by mechanical stress has already been described in various tissues [31,32] and particularly in articular cartilage subjected to dynamic compression representative of the physiological range [6].

Regulation of COX-2 mRNA expression in cartilage by mechanical stress has already been reported in the literature [6]. Notably, elements including AP-1 sites, cyclic AMP response elements (CREs) and shear stress response elements (SSRE) are found in the promoter region of mechanical stress-response genes, such as those encoding COX-2 and inducible NO synthase. Shear stress response elements contain a TPA response element to which NFκB, which is part of a main mechanical pathway, binds [33]. Ogasawara and colleagues [34] have described the role of C/EBP beta, AP-1 sites and CREB in shear stress-induced COX-2 expression in osteoblasts. Moreover, post-transcriptional regulation by mRNA stabilization seems to be involved in COX-2 gene expression in vascular endothelial cells subjected to fluid shear stress [35]. In addition to these studies at the mRNA level, we show here, for the first time in cartilage, that COX-2 is also increased at the protein level.

Interestingly, our data indicate that COX-3, also named COX-1 V1, is expressed in mouse costal cartilage. COX-3, which was cloned in 2002, was derived from COX-1 through retention of intron 1 in its mRNA. This probably resulted in the modification of the active site conformation of the enzyme. COX-3 expression has actually been found in several canine, human and rodent tissues, but never in cartilage, whatever the species [18]. In this present study, we report for the first time the expression of COX-3 (mRNA and protein) in mouse cartilage. Moreover, we show that mechanical loading did not modify its expression. As COX-1 and COX-3 are derived from the same gene, these enzymes share the same promoter. However, no sites that are regulated through mechanical stress or pro-inflammatory cytokines have been found so far in the COX-1 promoter, which is consistent with the fact that COX-1 is constitutively and ubiquitously expressed. Thus, this might explain the lack of COX-3 regulation by compression.

The regulation of mPGES-2 expression has never been described in cartilage. mPGES-2 is ubiquitously expressed under basal conditions in many tissues and is activated by reducing agents, but its role in PGE_2 _release in both basal and inflammatory contexts remains unclear. In human rheumatoid synoviocytes, expression of mPGES-1 increased with severity of the disease, whereas that of mPGES-2 did not [23]. In COX-2-deficient mouse brains, a decreased release of PGE_2 _and a decreased expression of mPGES-2, but not of mPGES-1 or cPGES, was observed, suggesting that mPGES-2 could be functionally coupled to COX-2 [36]. In the present study, mPGES-2 expression was similar in compressed and uncompressed cartilage explants, suggesting that mPGES-2 is not encoded by a mechanosensitive gene.

The striking point of our study is the evidence that mPGES-1 is encoded by a mechanosensitive gene. In recent years, several studies have demonstrated that inflammation induces mPGES-1. In rat paws of the acute and chronic arthritis model, up-regulation of mPGES-1 mRNA and protein expression was observed. Moreover, levels of mPGES-1 mRNA and protein were markedly elevated in OA versus normal cartilage [37]. Additionally, we and others have previously reported an overexpression of mPGES-1 in OA chondrocytes in primary cultures stimulated by IL-1 [21,22]. Interestingly, our results identify an earlier significant transcriptional expression (as soon as 2 hours) after mechanical stress compared to the effect of IL-1 (after 12 hours). Moreover, its induction was higher with compression (five-fold) compared to IL-1 stimulation (three-fold). A structural comparison of COX-2 and mPGES-1 promoters revealed that the gene encoding mPGES-1 does not contain transcriptional elements that are classically involved in response to mechanical stress (AP-1, CRE, SSRE), as described for the COX-2 promoter [38]. It suggests that divergent transcriptional mechanisms are responsible for inducible mPGES-1 regulation. Yokota and colleagues [39] have described the role of the nuclear regulator CITED2 (CBP/p300-interacting transactivator with ED-rich tail 2) in shear-induced down-regulation of matrix metalloproteinase 1 and matrix metalloproteinase 13. This mechanism could also occur in compression-induced mPGES-1 expression. But post-transcriptional regulation could also be present since a stabilization of mPGES-1 mRNA has been recently described in cardiomyocytes, leading to delayed protein expression via cJNK [40].

Our data suggest that loading triggers mPGES-1 and COX-2 colocalization in the perinuclear region of the chondrocytes. This result is in line with the study from Kojima and colleagues [22] that recently described COX-2 and mPGES-1 colocalization around the nuclei of chondrocytes after IL-1 stimulation.

Selective inhibitors of mPGES-1 have been developed recently [41]. Effectively, targeting the mPGES-1 enzyme in preference to COX-2 should represent a new therapeutical approach to treating joint diseases such as OA. Interestingly, Cheng and colleagues [42] reported that mPGES-1-null mice exhibit no alteration in thrombogenesis or blood pressure, whereas selective COX-2 inhibitors (Coxib) did. These results suggest that inhibitors of mPGES-1 may retain their anti-inflammatory efficacy by depressing PGE_2_, while avoiding the adverse cardiovascular consequences associated with COX-2 deletion [42].

To understand the weak PGE_2 _release at 18 and 24 hours after compression, we focused on 15-PGDH mRNA expression because 15-PGDH is one of the major PGE_2_-inactivating enzymes [43]. 15-PGDH expression decreases in response to IL-1β treatment in trophoblast cells in primary culture [44] as well as in response to lipopolysaccharide (LPS) treatment in liver and lungs of rats [45]. Moreover, 15-PGDH expression is reduced in inflammatory bowel disease [46]. To our knowledge, 15-PGDH expression in cartilage has never been reported and its expression under mechanical stress was unknown. The findings presented in this study first indicate that 15-PGDH is expressed in mouse cartilage and is induced after 4 hours of compression (three-fold induction). Genomic structural analysis revealed that the human 15-PGDH promoter contains binding sites for AP-1 and CREB, two transcriptional factors implicated in the regulation of gene expression subjected to mechanical stress [47]. We suggest that the 15-PGDH enzyme could be implicated in the catabolism of PGE_2 _after 18 hours. So, proinflammatory stimuli (LPS or cytokines) induce monophasic PGE_2 _synthesis whereas mechanical stress triggers a biphasic response in cartilage, PGE_2 _synthesis followed by PGE_2 _degradation. We hypothesize that this response corresponds to an adaptation of the tissue to this stress.

The choice of an experimental model to study the physiological regulation of cartilage homeostasis by mechanical stress is challenging. Three types of mechanical stress have been described: shear stress, strain and compressive stress. Overloading of the joints, a major risk factor for OA, mainly induces compressive stress. However, shear stress and strain have also been described when overloading is experimentally applied on cartilage. The compression of chondrocytes embedded in agarose or collagen gels as well as the compression of *ex vivo *porcine cartilage explants represent the main strategies developed [6,16,26,48]. Among the literature, it appears obvious that the consequences to metabolism of chondrocytes vary according to the type of stress applied. Takahashi and colleagues [49] reported that static compression promotes type II collagen, whereas cyclic loading could denature type II collagen in articular chondrocytes [50]. Here, we used mouse cartilage explants subjected to cyclical uniaxial mechanical stress. Mouse cartilage was chosen because of the availability of a large choice of biomolecular tools suitable to study it, such as genetically modified mice. However, many labs work on discs of porcine or bovine cartilage, permitting a more homogeneous compressive stress. In our model, the compressive stress is less uniform because of the size of the explants. Enhancements to our system, in order to better define the stress applied, is ongoing.

We decided to work on costal cartilage rather than articular cartilage because of the extremely small quantity of the latter available. However, we performed comparative experiments in order to validate our model with costal cartilage explants. We observed an induction of PGE_2 _release in both types of cartilage, but with slight differences in kinetics.

The mechanoreceptor integrin α5β1 is considered by many authors to be the critical mechanoreceptor for transducing signal from the extracellular matrix to the chondrocyte [8]. Our data confirm the major role of this receptor in compression-induced PGE_2 _release.

Among the mechanotransduction pathways recently described, Mitogen-Activated Protein Kinase (MAPK) plays a major role. In smooth muscle cells and fibroblasts, mechanical strain increases the activity of all three MAPKs, namely p38 MAPK, ERK 1/2 and Junk kinase [51,52]. In particular, *ex vivo *cartilage compression has been reported to activate the three MAPKs, but with different kinetics [9]. Preliminary results suggest that this is also the case in our model (personal communication).

Surprisingly, overexpression of COX-2 and mPGES-1 proteins was observed at 18 hours in uncompressed samples. Several authors have described the release of mediators, such as basic fibroblast growth factor (bFGF) and IL-1α, in cartilage after explantation and cutting [53,54]. Moreover, Fermor and colleagues [6] reported that uncompressed explants of articular cartilage exhibited a significant production of PGE_2 _(from 14 pg/mg/ml at 24 hours after explantation to 1 pg/mg/ml at 72 hours). We have considered that dissection alone induces COX-2 and mPGES-1 protein expression in the uncompressed samples.

As mature articular cartilage is an avascular tissue, the oxygen supply to resident chondrocytes could rather limit and influence chondrocyte activation under a mechanical signal. Fermor and colleagues have shown that oxygen tension influences the endogenous production of NO and PGE_2 _in porcine cartilage explants in response to mechanical stimulation. Under mechanical compression, PGE_2 _production in cartilage at 20% O_2 _increased 50-fold, but in cartilage at 5% O_2 _it increased only 4-fold and in cartilage at 1% O_2 _it did not increase at all [48]. Since previous studies have suggested that the oxygen tension in the superficial layer of articular cartilage is higher than in the deeper layer [55], our results could have occurred, at least in part, in the superficial zone of articular cartilage.

## Conclusion

We have demonstrated that COX-2 and mPGES-1 are mechanosensitive enzymes after dynamic compression. Moreover, the key enzyme implicated in the catabolism of PGE_2_, 15-PGDH, is expressed in cartilage and its expression is regulated by compression. As mPGES-1 is currently described as a potential therapeutic target for controlling PGE_2 _release in OA, further research into the regulation of this mechanosensitive gene should be helpful for a better understanding of the key pathways implicated in its expression in order to counteract cartilage degradation in overloading joints.

## Abbreviations

15-PGDH = NAD+-dependent 15 hydroxy prostaglandin dehydrogenase; BSA = bovine serum albumin; C/EBP = CAAT enhancer binding protein (C/EBP); COX = cyclooxygenase; cPGES = cytosolic PGES; CRE = cyclic AMP response element; CREB = cyclic AMP response element-binding protein; ERK = extracellular signal regulated kinases; FGF = fibroblast growth factor; HPRT = hypoxanthine-guanine phosphoribosyltransferase; IL = interleukin; IL1-Ra = inteleukin 1 receptor antagonist; JNK = c-jun-N-terminal kinase; LPS = lipopolysaccharides; MAPK = mitogen-associated protein kinase; mPGES = microsomal PGES; NFkB = nuclear factor kappa-B; NO = nitric oxide; OA = osteoarthritis; PBS = phosphate-buffered saline;; PGE^2^ = prostaglandin E2; PGES = prostaglandin E synthase; RT-PCR = reverse transcription PCR; SEM = standard error of the mean; SSRE = shear stress response element.

## Competing interests

The authors declare that they have no competing interests.

## Authors' contributions

MG performed animal surgery, compression experiments, PGE_2 _and NO assays, PCR experiments, western blotting experiments and immunohistochemistry experiments, analyzed the results and drafted the manuscript. FB was involved in the conception and design of the study and critically revised the manuscript for important intellectual content. AL performed animal surgery, compression experiments, PGE_2 _and NO assays and western blotting experiments. AP performed animal surgery, compression experiments and PGE_2 _and NO assays. ST initiated and participated in compression experiments. JLS performed immunohistochemistry experiments. CJ designed the study, participated in data analysis and helped draft the manuscript. All authors read and approved the final manuscript.

## Figures and Tables

**Table 1:** Primer sequences used to detect mRNA in mouse costal cartilage explants

Genes	Temperature (°C)	Genbank ID	Forward primer	Reverse primer	Amplicon length (bp)
HPRT	58	NM_013556	5'-gctggtgaaaaggacctct-3'	5'-cacaggactagaacacctgc-3'	249
COX-1	58	BC005573	5'-ctttgcacaacacttcacccacc-3'	5'-agcaacccaaacacctcctgg-3'	285
COX-2	58	NM_011198	5'-gcattctttgcccagcactt-3'	5'-agaccaggcaccgaccaaaga-3'	299
COX-3	58	AY547265	5'-tgaacgctaggctcaactctc-3'	5'-ggttctggcacggatagtaac-3'	349
cPGES	58	AY281130	5'-agtcatggcctaggttaac-3'	5'-tgtgaatcatcatctgctcc-3'	196
mPGES-1	58	NM_022415	5'-ctgctggtcatcaagatgtacg-3'	5'-cccaggtaggccacgtgtgt-3'	294
mPGES-2	58	BC004846	5'-aagacatgtcccttctgc-3'	5'-ccaagatgggcactttcc-3'	133
15-PGDH	58	NM_008278	5'-gccaaggtagcattggtggat-3'	5'-cttccgaaatggtctacaact-3'	164

15-PGDH, NAD+-dependent 15 hydroxy prostaglandin dehydrogenase; COX, cyclooxygenase; HPRT, hypoxanthine-guanine phosphoribosyltransferase; cPGES, cytosolic PGES; mPGES, microsomal PGES; PGES, prostaglandin E synthase.
